# Supplementary material for: Afadin-deficient mouse retinas exhibit severe neuronal lamination defects but preserve visual functions
Source: eLife. 2025 Dec 22;14:RP105627. doi: 10.7554/eLife.105627 (PMC12721710; doi:10.7554/eLife.105627)
Supplement: Figure 2—figure supplement 1—source data 1. [file elife-105627-fig2-figsupp1-data1.zip › Figure 2-figure supplement 2, Source Data 1 .pdf]

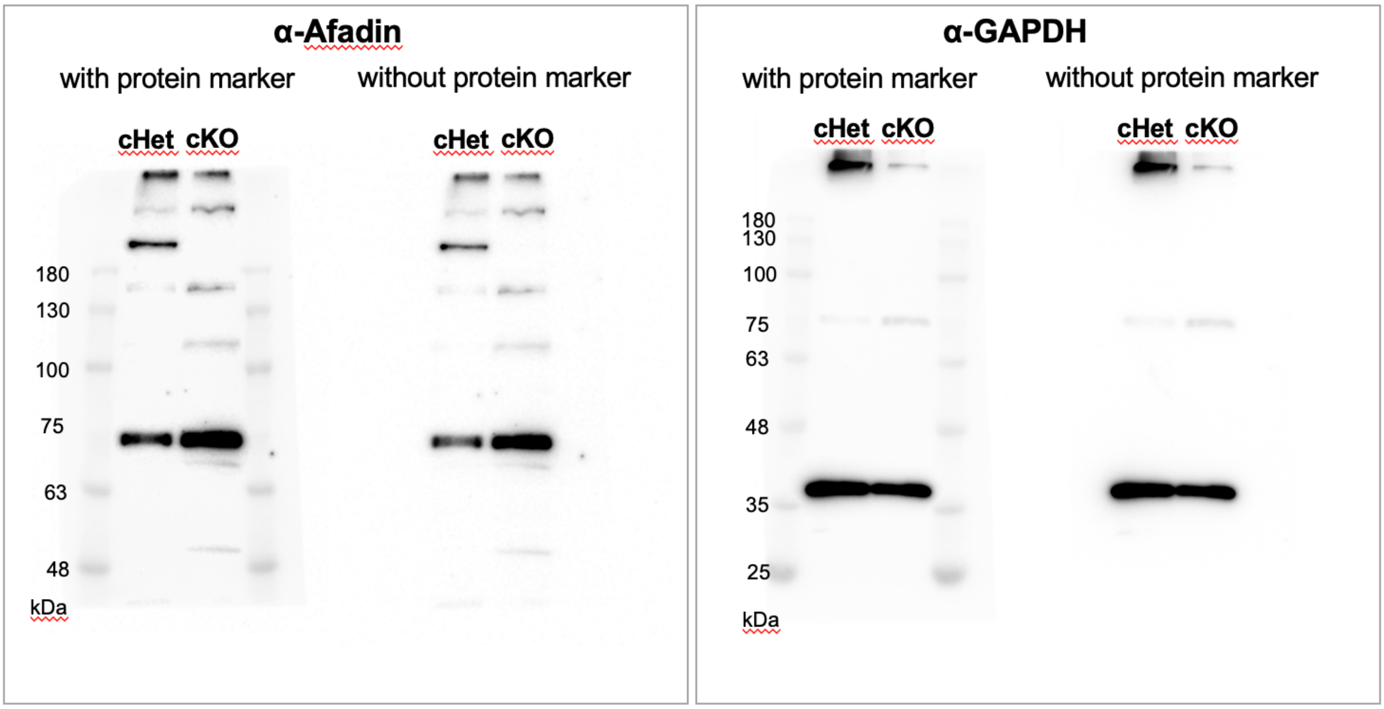

Figure 2-figure supplement 2-Source Data 1. Original membranes corresponding to Figure 2-figure supplement 1C. BlueStar Prestained Protein Marker (#NE-MWP03) were employed. The images of the protein marker and chemiluminescent signal were merged in each left panel.
