# Supplementary figures and images for: Afadin-deficient mouse retinas exhibit severe neuronal lamination defects but preserve visual functions
Source: eLife. 2025 Dec 22;14:RP105627. doi: 10.7554/eLife.105627 (PMC12721710; doi:10.7554/eLife.105627)

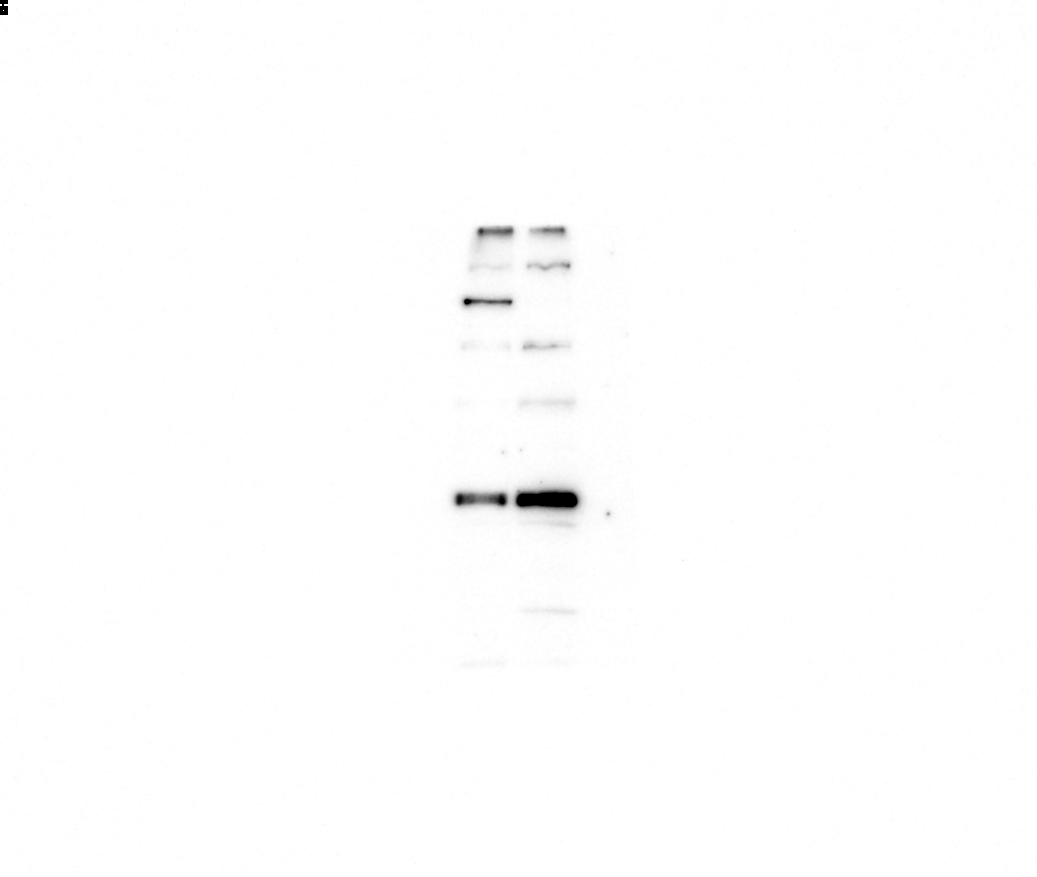

Supplement: Figure 2—figure supplement 1—source data 2. [file elife-105627-fig2-figsupp1-data2.zip › Figure 2-figure suplement 1-Source data 2/241017 Afadin Het cKO ╬▒-Afadin WB image with protein marker.tif]

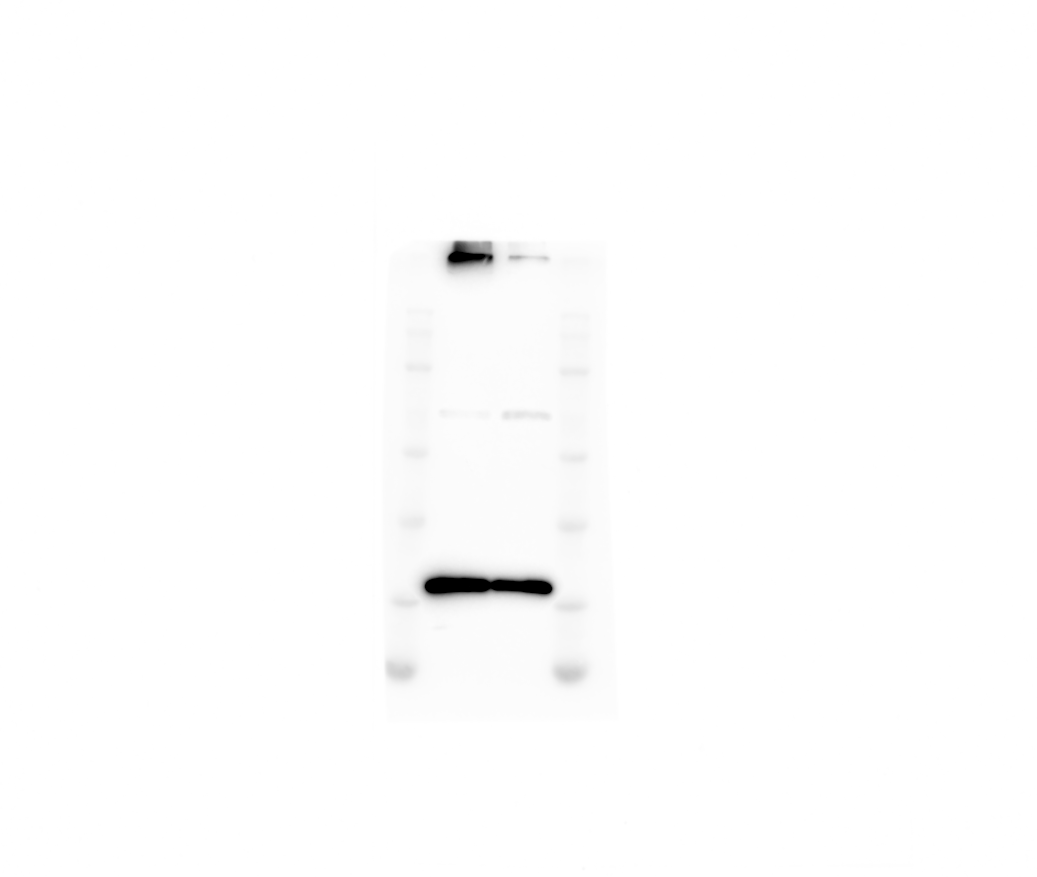

Supplement: Figure 2—figure supplement 1—source data 2. [file elife-105627-fig2-figsupp1-data2.zip › Figure 2-figure suplement 1-Source data 2/241017 Afadin Het cKO ╬▒-GAPDH WB with marker.tif]

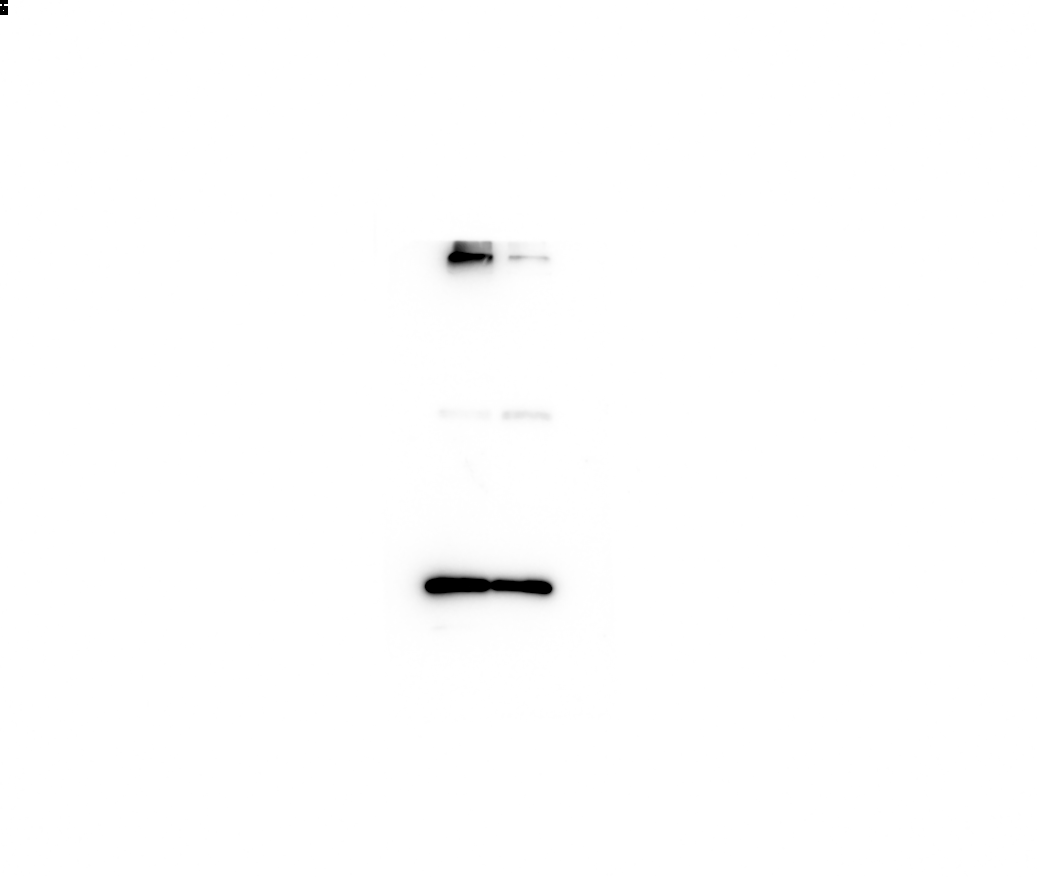

Supplement: Figure 2—figure supplement 1—source data 2. [file elife-105627-fig2-figsupp1-data2.zip › Figure 2-figure suplement 1-Source data 2/241017 Afadin Het cKO ╬▒-GAPDH WB.tif]

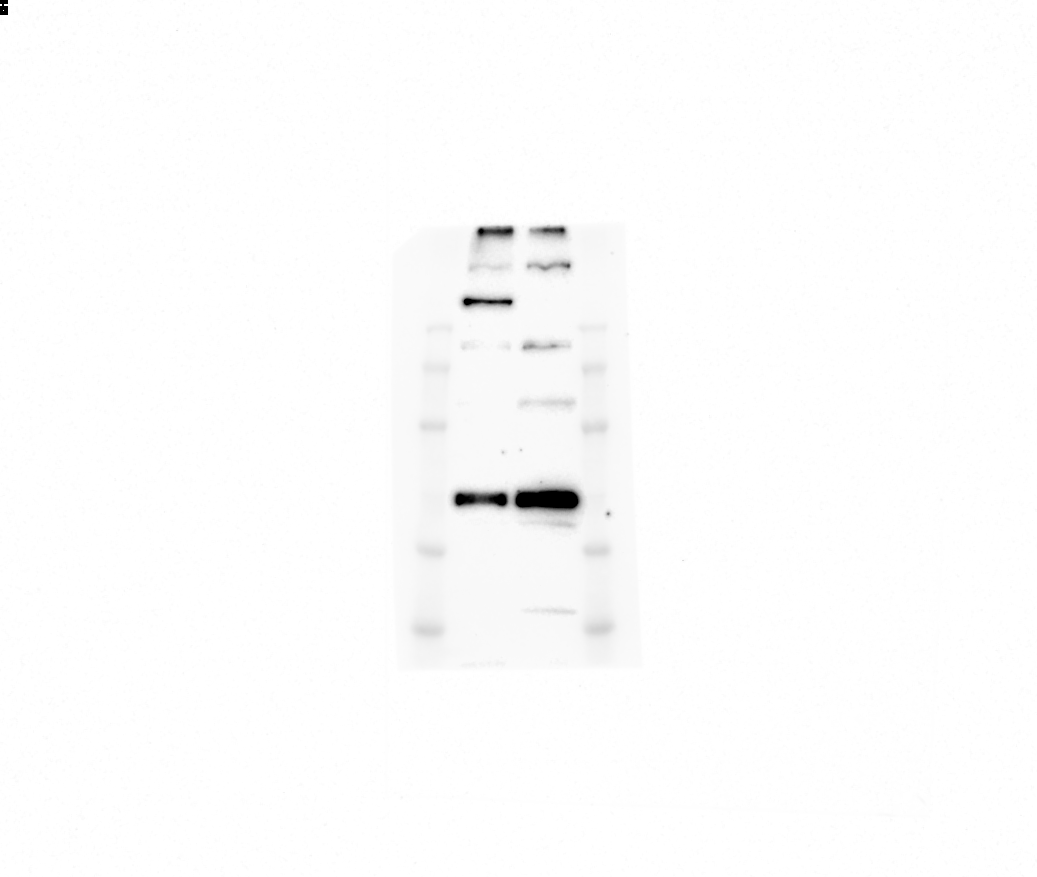

Supplement: Figure 2—figure supplement 1—source data 2. [file elife-105627-fig2-figsupp1-data2.zip › Figure 2-figure suplement 1-Source data 2/241017 Afadin Het cKO ╬▒-Afadin WB.tif]
