# Supplementary material for: Afadin-deficient mouse retinas exhibit severe neuronal lamination defects but preserve visual functions
Source: eLife. 2025 Dec 22;14:RP105627. doi: 10.7554/eLife.105627 (PMC12721710; doi:10.7554/eLife.105627)
Supplement: Supplementary file 1. [file elife-105627-supp1.docx]

| **Primer name** | **Sequence (5' to 3')** |
| --- | --- |
| Opnsw Fw | CAGCATCCGCTTCAACTCCAA |
| Opnsw Rv | GCAGATGAGGGAAAGAGGAATGA |
| Opnmw Fw | CTCTGCTACCTCCAAGTGTGG |
| Opnmw Rv | AAGTATAGGGTCCCCAGCAGA |
| Nrl Fw | GCTGTGCCTTTCTGGTTCTGA |
| Nrl Rv | GCTCCCGCTTTATTTCGAACT |
| Rho Fw | GACTCTGCCAGCTTTCTTTGCT |
| Rho Rv | GCGTCGTCATCTCCCAGTGGA |
| Trpm1 Fw | ATGCGCCCATTGTCAAGTTC |
| Trpm1 Rv | TTCTCCAATGCAAGGCTCACA |
| Grm6 Fw | GTCCATCATGGTCGCCAATGT |
| Grm6 Rv | AGTCATAGCGTGTGGAGTCAC |
| Chx10 Fw | GGCGACACAGGACAATCTTTA |
| Chx10 Rv | TTCCGGCAGCTCCGTTTTC |

**Supplementary File 1 The primer sequences used for RT-qPCR**
